# IL-6 and INF-γ levels in patients with brucellosis in severe epidemic region, Xinjiang, China

**DOI:** 10.1186/s40249-020-00666-7

**Published:** 2020-05-07

**Authors:** Zhi-Qiang Lin, Guo-Yue Lin, Wen-Wen He, Chi Zhang, Rui Zhang, Yuan-Da Li, Fan Wang, Ying Qin, Li Duan, Dou-Dou Zhao, Xiao-Juan Qu, Hui Gao, Hai Jiang

**Affiliations:** 1 Department of Clinical Laboratory, Sixth People’s Hospital, Urumqi, Xinjiang, Uygur Autonomous Region China; 2 State Key Laboratory for Infectious Disease Prevention and Control, Collaborative Innovation Center for Diagnosis and Treatment of Infectious Diseases, National Institute for Communicable Disease Control and Prevention, Chinese Center for Disease Control and Prevention, Beijing, China

**Keywords:** Brucellosis, Cytokines, Immunity, IL-6, INF-γ

## Abstract

**Background:**

The incidence of brucellosis, which is caused by the *Brucella* species of bacteria, is rapidly rising worldwide; however, few studies have investigated the immune response to this pathogen and clinical biochemical features. In this paper, we examined the levels of various cytokines and inflammatory factors as well as clinical course characteristics in patients with brucellosis, in order to provide evidence for the diagnosis, assessment, and prognosis of this infectious disease.

**Methods:**

A total of 191 brucellosis inpatients (50 acute cases and 141 chronic cases), as well as 60 healthy control subjects, were included in the analysis. We investigated changes in the levels of six cytokines (IL-4, IL-6, IL-10, IL-17, TNF-α, INF-γ) and related clinical biochemical markers in patients with acute and chronic brucellosis in Xinjiang, China. Possible factors were statistically analyzed using the *t* test, *χ*^*2*^ test, z test and a multivariate logistic stepwise regression test.

**Results:**

We found that IL-4, IL-6, IL-10, IL-17, IFN-γ, and TNF-α levels were higher in those with brucellosis than in controls (*P* <  0.05). With regard to disease progression, procalcitonin (PCT) and C-reactive protein (CRP) levels were significantly higher in those with an acute infection compared to chronic cases (*P* <  0.05). We found that the expression of all six cytokines tested was closely related to the degree of brucellosis using univariate logistic regression; however, only IL-6 and INF-γ levels were independent factors associated with the severity of brucellosis.

**Conclusions:**

Assessing cytokine levels in patients with acute and chronic brucellosis is not only useful for detecting the immune response, but can also be indicative of the severity of brucellosis. In particular, we propose IL-6 and INF-γ levels may be useful independent predictive factors in the clinical evaluation and diagnosis of brucellosis.

## Background

Brucellosis is a contagious allergic disease caused by the *Brucella* species of bacteria that is classified as a Class B infectious disease according to the Law of the People’s Republic of China on the Prevention and Control of Infectious Diseases [1, 2]. Globally, there are 500 000 new cases of brucellosis each year, and the true incidence is always much higher than the reported number of cases [3]. In China, the number of cases of brucellosis has rapidly risen since the mid-1990s [4], and it has become one of the fastest-growing infectious diseases [5]. Brucellosis infection has a chronic disease course, and unfortunately, there is currently no effective human vaccine [6, 7].

Brucellosis is widely distributed, with epidemic areas mainly occurring in frontier grassland pastoral areas, where both people and animals are afflicted. Insufficient environmental hygiene, disease prevention measures, and medical conditions often lead to the occurrence, spread, and prolongation of brucellosis [8]. The main epidemic areas of brucellosis in China comprise five pastoral locations: Xinjiang, Inner Mongolia, Qinghai, Ningxia, and Tibet. Xinjiang is a multi-ethnic animal husbandry area, and the annual incidence of brucellosis in high-risk professional populations (farmers, herdsmen and veterinarians) is about 6.4–7.6% [9]. To date, research on brucellosis in China has mainly focused on epidemiological investigations [10, 11], with only a few studies investigating the cytokine immune response to this pathogen. In particular, Han et al. [12] reported that high levels of the cytokine interferon (IFN-γ) may be a characteristic of brucellosis chronicity. However, the relationships between the various cytokines and inflammatory factors and the clinical course of brucellosis have not yet been reported.

In this paper, we examined the levels of various cytokines and inflammatory factors as well as clinical course characteristics in patients with brucellosis, in order to provide evidence for the diagnosis, assessment, and prognosis of this infectious disease.

## Methods

### Study design and subjects

This was a retrospective study of 191 brucellosis patients selected in Sixth People’s Hospital of Urumqi from August 2017 to November 2018, including 50 acute cases (less than three months) and 141 chronic cases (more than six months). Patients with a clear clinical history and experimental diagnosis were included. Sixty healthy residents living in the affected areas were selected as the control group. All subjects provided informed consent to participate in the study. Diagnostic criteria were in strict accordance with the “Diagnostic Criteria for Brucellosis (WS 269-2019)” ([13], Supplementary material).

### Specimen collection

A total of 3–4 ml of venous blood was collected and centrifuged at 1200×*g* for 5 min. The serum was then removed and stored at a temperature range of 4 °C to − 20 °C prior to testing.

### Cytokine detection

Using a FACS Calibur flow cytometer (BD, San Diego, USA), we detected six cytokines (IL-4, IL-6, IL-10, IL-17, IFN-γ, TNF-α) by using a cytokine detection kit (multiple microsphere flow immunofluorescence method; Qingdao Ruisikeer Biotechnology Co., Ltd. Operation, Qingdao, China) as per the manufacturer’s instructions.

### Biochemical indicators

Patients were tested for brucellosis using the rose bengal plate agglutination test, serum agglutination test, and blood culture. We used a Roche Cobas e601 automatic analyzer (Roche, Shanghai, China,) for electrochemiluminescence detection of procalcitonin (PCT), a Siemens ADVIA 2400 automatic analyzer latex (Siemens, Shanghai, China) for enhanced immunoturbidimetric detection of hypersensitive C-reactive protein (CRP), and a Roche Cocha 8000 c701 automatic analyzer (Roche, Shanghai, China) for the immunoturbidimetric detection of rheumatoid factor (RF). We also used an automatic dynamic erythrocyte sedimentation apparatus (model: ORON-200) to detect the erythrocyte sedimentation rate (ESR). Finally, *Brucella* infection was detected using the BacT/ALERT 3D automatic bacterial/mycobacterial culture detection system. All methods were carried out according to the manufacturer’s instructions.

### Data analysis

Statistical analysis was performed using Stata 15.0 (Stata Corp LP, College Station, TX, USA) software. Possible factors were statistically analyzed using the *t* test, *χ*^*2*^ test and a multivariate logistic stepwise regression test. A *P*-value of < 0.05 was considered statistically significant.

## Results

### Demographic data and clinical characteristics of the brucellosis patients

The main demographic features of 191 brucellosis cases were shown in Table 1. Brucellosis patients were mostly male, with the male:female ratio being 2.35. A total of 96 patients (50.2%) were aged between 31 and 50 years. There were 84 cases (44.0%) of Uygur nationality. Of the 191 patients with brucellosis, 140 were farmers and herdsmen.

**Table 1:** Demographic features of 191 brucellosis cases

Demographic features	Number (%)
**Sex**	
Male	134 (70.2%)
Female	57 (29.8%)
**Age group (years)**	
< 20	12 (6.3%)
20–30	23 (12.0%)
31–40	40 (20.9%)
41–50	56 (29.3%)
51–60	38 (19.9%)
61–70	22 (11.5%)
**Nationality**	
Uygur	84 (44.0%)
Ha	44 (23.0%)
Han	43 (22.5%)
Hui	11 (5.8%)
Others	9 (4.7%)
**Occupation**	
Farmer & herdsman	140 (73.3%)
Veterinarians	21 (10.1%)
Others	30 (15.7%)

Among the 191 patients with brucellosis, 23 (46.0%) patients in the acute group and 89 (63.1%) in the chronic group had brucellosis arthritis; 23 (46.0%) patients in the acute group and 76 (54.0%) in the chronic group had lumbar disc hyperplasia; 11 (22.0%) patients in the acute group and 20 (14.2%) in the chronic group had cholecystitis; and 4 (8.0%) patients in the acute group and 9 (6.4%) in the chronic group had orchitis. Levels of IL-4, IL-6, IL-10, IL-17, IFN-γ, and TNF-α were all significantly higher in those with brucellosis than in the control group (all *P* <  0.05; Table 2).

**Table 2:** Comparison of cytokine levels in patients with brucellosis and controls

Clinical indicators	Case group (*n* = 191)	Control group (*n* = 60)	*t*/*χ*^2^/z	*P-*value
Age (years), mean ± *SD*	43 ± 14	40 ± 11	1.518 (*t*)	0.130
Gender, *n* (%)	137 (70.2)	40 (66.7)	0.609 (*χ*^2^)	0.609
IL-4 (pg/ml)	2.74 (0.97–6.25)	0.56 (0.53–0.64)	8.379 (z)	< 0.001
IL-6 (pg/ml)	34.10 (13.05–60.8)	0.78 (0.41–2.52)	7.183 (z)	< 0.001
IL-10 (pg/ml)	1.83 (1.15–2.75)	0.89 (0.77–1.13)	4.857 (z)	< 0.001
IL-17 (pg/ml)	6.77 (2.48–13.74)	1.03 (0.68–1.20)	7.234 (z)	< 0.001
TNF-α (pg/ml)	35.23 (5.79–74.98)	1.33 (0.80–2.17)	6.264 (z)	< 0.001
INF-γ (pg/ml)	212.96 (42.30–436.72)	1.69 (1.36–1.89)	7.683 (z)	< 0.001

All data are expressed as mean (interquartile range), unless otherwise indicated

### Changes in cytokine levels in patients with acute and chronic brucellosis

IL-6, IL-10, IL-17, IFN-γ, and TNF-α levels were significantly higher in the acute group than in the chronic group (*P* <  0.05; Table 3). We also found that the blood culture positive rate was significantly higher in the acute group than in the chronic group (*P* <  0.001), and PCT and CRP expression levels were significantly higher in the acute group than in the chronic group (*P* <  0.001), as shown in Table 3.

**Table 3:** Comparison of cytokine levels in patients with acute and chronic brucellosis

Clinical indicators	Acute group (*n* = 50)	Chronic group (*n* = 141)	*t*/*χ*^2^/z	*P-*value
Age (years), mean ± *SD*	40 ± 14	44 ± 14	1.813(*t*)	0.071
Gender, *n* (%)	36 (72.0)	98 (69.5)	0.740 (*χ*^2^)	0.109
Brucellosis arthritis, *n* (%)	23 (46.0)	89 (63.1)	4.460 (*χ*^2^)	0.035
Lumbar disc hyperplasia, *n* (%)	23 (46.0)	76 (54.0)	0.922 (*χ*^2^)	0.337
Chronic cholecystitis, *n* (%)	11 (22.0)	20 (14.2)	1.658 (*χ*^2^)	0.198
Orchitis, *n* (%)	4 (8.0)	9 (6.4)	0.152 (*χ*^2^)	0.696
Blood culture, positive, *n* (%)	20 (40.0)	23 (16.5)	11.508 (z)	< 0.001
RF (IU/ml)	7.2 (3.8–9.9)	6.2 (3.7–9.8)	0.633 (z)	0.526
ESR (mm/h)	25 (15–40)	17 (7–37)	2.040 (z)	< 0.041
anti-O (IU/ml)	79 (33–137)	87 (45–182)	0.717 (z)	0.473
PCT (pg/ml)	0.08 (0.06–0.12)	0.04 (0.03–0.06)	3.914 (z)	< 0.001
CRP (mg/L)	12.6 (5.0–30.8)	3.3 (1.2–16.2)	3.931 (z)	< 0.001
IL-4 (pg/ml)	4.03 (1.29–8.08)	2.02 (0.96–5.90)	1.874 (z)	0.061
IL-6 (pg/ml)	65.32 (36.27–68.60)	29.38 (11.30–53.09)	3.683 (z)	< 0.001
IL-10 (pg/ml)	2.38 (1.51–3.34)	0.89 (0.77–1.13)	3.350 (z)	< 0.001
IL-17 (pg/ml)	11.98 (3.92–16.49)	5.27 (2.24–12.49)	2.856 (z)	< 0.004
TNF-α (pg/ml)	50.88 (16.59–96.69)	28.24 (5.30–64.40)	2.387 (z)	< 0.017
INF-γ (pg/ml)	319 (145.14–588.52)	160.52 (36.59–418.54)	2.979 (z)	< 0.003

All data are expressed as mean (interquartile range), unless otherwise indicated

*CRP* C-reactive protein, *ESR* Erythrocyte sedimentation rate, *PCT* Procalcitonin, *RF* Rheumatoid factor

### The relationship between antibody titer and cytokine levels

Patients were divided into a high titer group (> 1:100, *n* = 79) and a low titer group (≤ 1:100, *n* = 112) according to the diagnostic level of the brucellosis antibody titer. We found that the blood culture positive rate and ESR, as well as the levels of RF, PCT, and CRP, were significantly higher in the high titer group (*P* <  0.001; Table 4). However, there was no difference in the levels of anti-O between the two groups. In addition, IL-6 levels were significantly higher in the high antibody titer group than the low antibody titer group (*P* <  0.001; Table 4), but all other cytokine levels were similar.

**Table 4:** Relationship between *Brucella* antibody titer and cytokine levels

Clinical indicators	Antibody titer ≤1:100 (*n* = 112)	Antibody titer > 1:100 (*n* = 79)	*z*	*P-*value
Blood culture, positive, *n* (%)	17 (15.3)	26 (33.3)	8.462	< 0.004
RF (IU/ml)	3.0 (0.8–9.7)	13.6 (3.0–31.4)	4.496	< 0.001
ESR (mm/h)	15 (7–31)	27 (15–46)	4.245	< 0.001
anti-O (IU/ml)	82.5 (44.5–156.5)	86.0 (36.0–185.0)	0.020	0.984
PCT (pg/ml)	0.04 (0.03–0.06)	0.07 (0.04–0.11)	2.372	< 0.017
CRP (mg/L)	3.03 (0.85–9.70)	13.65 (2.97–31.43)	4.496	< 0.001
IL-4 (pg/ml)	2.84 (0.98–6.16)	2.69 (0.97–6.25)	0.946	0.068
IL-6 (pg/ml)	30.46 (11.30–50.02)	46.20 (17.72–79.69)	2.840	< 0.004
IL-10 (pg/ml)	1.66 (1.14–2.52)	1.98 (1.15–3.13)	0.156	0.156
IL-17 (pg/ml)	6.54 (2.09–13.37)	6.77 (2.80–15.27)	0.942	0.346
TNF-α (pg/ml)	37.13 (6.05–61.89)	36.23 (5.79–86.44)	0.751	0.452
INF-γ (pg/ml)	183.115 (41.59–413.25)	270.91 (47.03–538.5)	1.620	0.105

All data are expressed as mean (interquartile range), unless otherwise indicated*CRP* C-reactive protein, *ESR* Erythrocyte sedimentation rate, *PCT* Procalcitonin, *RF* Rheumatoid factor

### The relationship between cytokine expression and brucellosis

We used logistic stepwise regression analysis to assess the relationship between the various cytokines and brucellosis. In our univariate analysis, IL-4, IL-6, IL-10, IL-17, IFN-γ, and TNF-α were risk factors for brucellosis (Table 5). In the multivariate logistic stepwise regression analysis, IL-6 and INF-γ were found to be independent risk factors for brucellosis after adjusting for age and gender.

**Table 5:** Multivariate logistic stepwise regression analysis of cytokine immunity and brucellosis

Variable		Single factor			Multiple factors	
	*OR* (95% *CI*)^a^	Wald	*P*-value	*OR* (95% *CI*)	Wald	*P*-value
IL-4	16.59 (3.583–76.826)	3.59	< 0.001			
IL-6	1.32 (1.142–1.547)	3.67	< 0.001	1.28 (1.012–1.056)	2.33	< 0.012
IL-10	4.793 (2.106–10.907)	3.74	< 0.001			
IL-17	11.14 (3.459–35.898)	4.04	< 0.001			
TNF-α	1.32 (1.080–1.635)	2.69	0.007			
INF-γ	1.268 (1.121–1.434)	3.78	< 0.001	1.29 (1.079–1.564)	2.01	< 0.006

^a^Odds ratio (*OR*) and 95% confidence interval (95% *CI*)

## Discussion

In this study, we examined the levels of six cytokines and inflammatory factors as well as clinical course characteristics of 191 patients with laboratory confirmed brucellosis. We found that IL-4, IL-6, IL-10, IL-17, IFN-γ, and TNF-α levels were higher in those with brucellosis than in controls. With regard to disease progression, PCT and CRP levels were significantly higher in those with an acute infection compared to chronic cases. The results from this study provide a basis for clinical diagnosis, assessment and case management of human brucellosis.

The gender and age distributions were consistent with previous studies [14, 15]. The primary transmission route of brucellosis was through occupational exposure (78%, 149/191), which is in accord with the results of epidemiological investigations in China [16, 17]. In other endemic countries, infections occur mostly due to ingestion of unpasteurized dairy products [18, 19]. In the Sixth People’s Hospital, many referred brucellosis patients were received. Therefore, most patients came to the clinic after several months of *Brucella* infection, when the disease had become chronic. The chronicity of *Brucella* infection may be related to its ability to escape the host’s immune response [20].

We found that age and gender were not related to the pathogenesis of acute and chronic brucellosis. However, the complications of brucellosis did vary; for example, the prevalence of spondylitis and arthritis was higher in those with chronic brucellosis than in those with acute infection. We also found that the positive rate of blood culture was significantly higher in the acute group, indicating that the disease course is related to the severity of bacterial infection, as well as the intensity of immune response. PCT and CRP levels were also higher in those with an acute infection. These are consistent with other reports done in China and Turkey [21, 22].

After entering the human body, *Brucella* likely activates immune cells and promotes the production of cytokines by T lymphocytes. We found that IL-4, IL-6, IL-10, IL-17, IFN-γ, and TNF-α levels were higher in patients with brucellosis than healthy controls, indicating a strong immune response was occurring. Indeed, previous studies have shown *Brucella* is effectively cleared through the secretion of IFN-γ, IL-2, and TNF-α by T helper (Th) 1 cells [23, 24].

We found that all cytokine levels were significantly higher in the acute group than in the chronic group, except for IL-4. IL-4 is the main cytokine released by Th2 cells to exert humoral immunity, which is not conducive to curing brucellosis [25].

With regards to the degree of infection (as assessed by brucellosis antibody titer), we found only IL-6 levels were altered. This indicates IL-6 is the main factor for the chronicization of brucellosis infection. In addition, the positive rate of blood culture and ESR, as well as the RF, PCT, and CRP levels, were elevated in those with high antibody titer, reflecting the development of the disease [26, 27].

Overall our findings indicate the acute immune response to *Brucella* infection is more intense than during the chronic stage of the disease. Our results differ from those reported by Han et al. [12], who found that IFN-γ levels were higher in chronic brucellosis than in acute brucellosis, and concluded that high IFN-γ levels may be predictive of brucellosis chronicity. This may be due to different methods for case classification. In this study, we examined the relationship between brucellosis and cytokine levels, and found IL-4, IL-6, IL-10, IL-17, IFN-γ, and TNF-α were all risk factors for brucellosis, although only IL-6 and INF-γ were independent risk factors for the severity of brucellosis. Finally, our results confirm previous data showing that Th1 cytokines help the body initially combat *Brucella*, while Th2 cytokines are involved as it progresses to brucellosis [28], although further research on the immune response to *Brucella* is warranted.

Our study was limited to the samples of brucellosis cases and controls. In the Sixth People’s Hospital, many referred brucellosis patients were received. Therefore, a relatively large chronic disease population has been included in the current study. In next steps, we are going to divided the 141 chronic cases into two refined groups, i.e., 6 to 12 months and more than 12 months, to better confirm the reliability of the results.

## Conclusions

Taken together, these findings demonstrate assessing cytokine levels in patients with brucellosis is not only useful for detecting the immune response, but can also be indicative of the severity of brucellosis. In particular, we propose IL-6 and INF-γ may be useful independent predictive factors in the clinical evaluation and diagnosis of brucellosis.

## Supplementary information

**Additional file 1.** Diagnostic Criteria for Brucellosis (WS 269–2019).

## Data Availability

Not applicable.
