# Supplementary material for: IL-6 and INF-γ levels in patients with brucellosis in severe epidemic region, Xinjiang, China
Source: Infect Dis Poverty. 2020 May 7;9:47. doi: 10.1186/s40249-020-00666-7 (PMC7203901; doi:10.1186/s40249-020-00666-7)
Supplement: Supplementary file 1 — Additional file 1. Diagnostic Criteria for Brucellosis (WS 269–2019). [file 40249_2020_666_MOESM1_ESM.docx]

The diagnostic criteria for brucellosis defined a clinical case of brucellosis as a patient presenting with a fever lasting several days or weeks accompanied with sweating; muscle and joint pain; fatigue; and/or symptoms such as hepatomegaly, splenomegaly, and lymph node enlargement. Confirmation relies on a positive result from one of the following diagnostic tests: culture and identification of *Brucella* spp. from clinical specimens or serological evidence of ≥1:100 titer by the serum agglutination test.
